# Aortic valve leaflet motion for diagnosis and classification of aortic stenosis using single view echocardiography

**DOI:** 10.1186/s44348-025-00051-8

**Published:** 2025-07-08

**Authors:** Thomas Meredith, Farhan Mohammed, Amy Pomeroy, Sebastiano Barbieri, Erik Meijering, Louisa Jorm, David Roy, Christopher Hayward, Jason C. Kovacic, David W. M. Muller, Michael P. Feneley, Mayooran Namasivayam

**Affiliations:** 1 Department of Cardiology, St Vincent’s Hospital, Sydney, NSW Australia; 2 Victor Chang Cardiac Research Institute, Sydney, NSW Australia; 3 University of New South Wales, Sydney, NSW Australia; 4 Centre for Big Data Research in Health, University of New South Wales, Sydney, NSW Australia; 5 Icahn School of Medicine at Mount Sinai, New York, NY USA; 6 Queensland Digital Health Centre, University of Queensland, Brisbane, QLD Australia

**Keywords:** Aortic valve stenosis, Echocardiography, Machine learning, Heart valve diseases

## Abstract

**Background:**

Accurate classification of aortic stenosis (AS) severity remains challenging despite detailed echocardiographic assessment. Adjudication of severity is informed by subjective interpretation of aortic leaflet motion from the first image parasternal long axis (PLAX) view, but quantitative metrics of leaflet motion currently do not exist. The objectives of the study were to echocardiographically quantify aortic leaflet motion using the PLAX view and correlate motion data with Doppler-derived hemodynamic indices of disease severity, and predict significant AS using these isolated motion data.

**Methods:**

PLAX loops from 200 patients with and without significant AS were analyzed. Linear and angular motion of the anterior (right coronary) leaflet were quantified and compared between severity grades. Three simple supervised machine learning classifiers were then trained to distinguish significant (moderate or worse) from nonsignificant AS and individual severity grades.

**Results:**

Linear and angular displacement demonstrated strong correlation with aortic valve area (r = 0.81 and r = 0.74, respectively). Severe AS cases demonstrated global leaflet motion of 2.1 mm, compared with 3.6 mm for moderate cases (*P* < 0.01) and 9.2 mm for control cases (*P* < 0.01). Severe cases demonstrated mean global angular rotation of 11°, significantly less than moderate (18°, *P* < 0.01) and normal cases (47°, *P* < 0.01). Using these novel metrics, a simple supervised machine learning model predicted significant AS with an accuracy of 90% and area under the receiver operator characteristics curve (AUC) of 0.96. Prediction of individual severity class was achieved with an accuracy of 72.5% and AUC of 0.88.

**Conclusions:**

Advancing severity of AS is associated with significantly reduced linear and angular leaflet displacement. Leaflet motion data can accurately classify AS using a single parasternal long axis view, without the need for hemodynamic or Doppler assessment. Our model, grounded in biological plausibility, simple linear algebra, and supervised machine learning, provides a highly explainable approach to disease identification and may hold significant clinical utility for the diagnosis and classification of AS.

**Supplementary Information:**

The online version contains supplementary material available at 10.1186/s44348-025-00051-8.

## Background

Aortic stenosis (AS) is one of the most common valvular heart conditions in developed countries [1]. Currently, the severity of AS is primarily determined using three Doppler-derived echocardiographic criteria: mean transvalvular pressure gradient, peak transvalvular velocity, and calculated aortic valve area (AVA) [2]. Despite detailed echocardiographic assessment, accurate classification of AS severity remains challenging. A significant proportion of patients demonstrate discordance between these criteria, and although several ancillary metrics of disease severity have been proposed, these add time, the possibility of alternate imaging, a lack of standardization of methods used to report AS severity, and cognitive burden to an already challenging process [3]. The parasternal long axis (PLAX) view, conventionally the first echocardiographic loop acquired, provides a wealth of information pertaining to cardiac function and left sided valvular pathology. Specific to AS, the PLAX view affords the analyst an immediate clue to the presence of AS based on the morphology and movement of the aortic valve. This important qualitative assessment sets the foundation for the next task: grading the severity of valvular restriction using Doppler-derived indices. There is presently no quantifiable measure of this visual assessment, nor does it inform diagnostic guidelines in a non-subjective manner.

Rapid advances in computer science have led to exciting applications of artificial intelligence to cardiac imaging [4–6]. Deep learning models deployed to PLAX images have demonstrated impressive diagnostic and classification accuracy in AS, without the need for any further image acquisition [7]. One drawback of black-box unsupervised approaches is the absence of granular explainability behind model decisions. Although it is unclear exactly why these deep learning approaches have arrived at a conclusion, inference from saliency heatmapping has revealed that important predictive features are being drawn from the aortic valve region [7], just as the human analyst does. Therefore, we hypothesized that (1) quantification of aortic leaflet motion from a single PLAX would be feasible, and would correlate with Doppler-derived hemodynamic indices of disease severity; (2) leaflet motion would demonstrate different patterns between gradient-based phenotypes of AS (i.e., severe high-gradient AS vs. severe low-gradient AS); and (3) quantification of leaflet motion from a single PLAX view would facilitate prediction of significant AS, without the need for additional image or Doppler acquisition.

## Methods

Based on pilot data, a sample size of 126 was calculated to provide 80% power (α = 0.05) to determine a statistical difference in mean angular displacement between control, moderate, and severe classes using Kruskal–Wallis rank sum testing and post hoc pairwise comparisons. We retrieved a random selection of 200 echocardiograms archived in the imaging database of St Vincent’s Hospital, Sydney, between 2014 and 2024 for patients with significant AS. Specifically, significant AS included moderate and severe grades, as adjudicated by the reporting cardiologist using contemporary guideline criteria [8]. A reference population without AS (normal echocardiograms) was also included. Patients with prosthetic aortic valves or bicuspid anatomy were excluded. Limited demographic data were extracted from each echocardiography report. Raw echocardiographic images were de-identified and conventional analysis was performed by an accredited cardiologist (TM) or cardiac sonographer (AP), in random order, at the Heart Valve Disease and Artificial Intelligence Laboratory, Victor Chang Cardiac Research Institute (Sydney, NSW, Australia). Conventional chamber measurements were performed according to contemporary American Society of Echocardiography guidelines [2]. Cases were classified by reporter-adjudicated severity as well as by strict Doppler hemodynamic criteria. Studies were inspected for quality, and the highest quality PLAX clips (zoomed or unzoomed, as available), were selected for leaflet analysis.

### Leaflet analysis

**Fig. 1:**
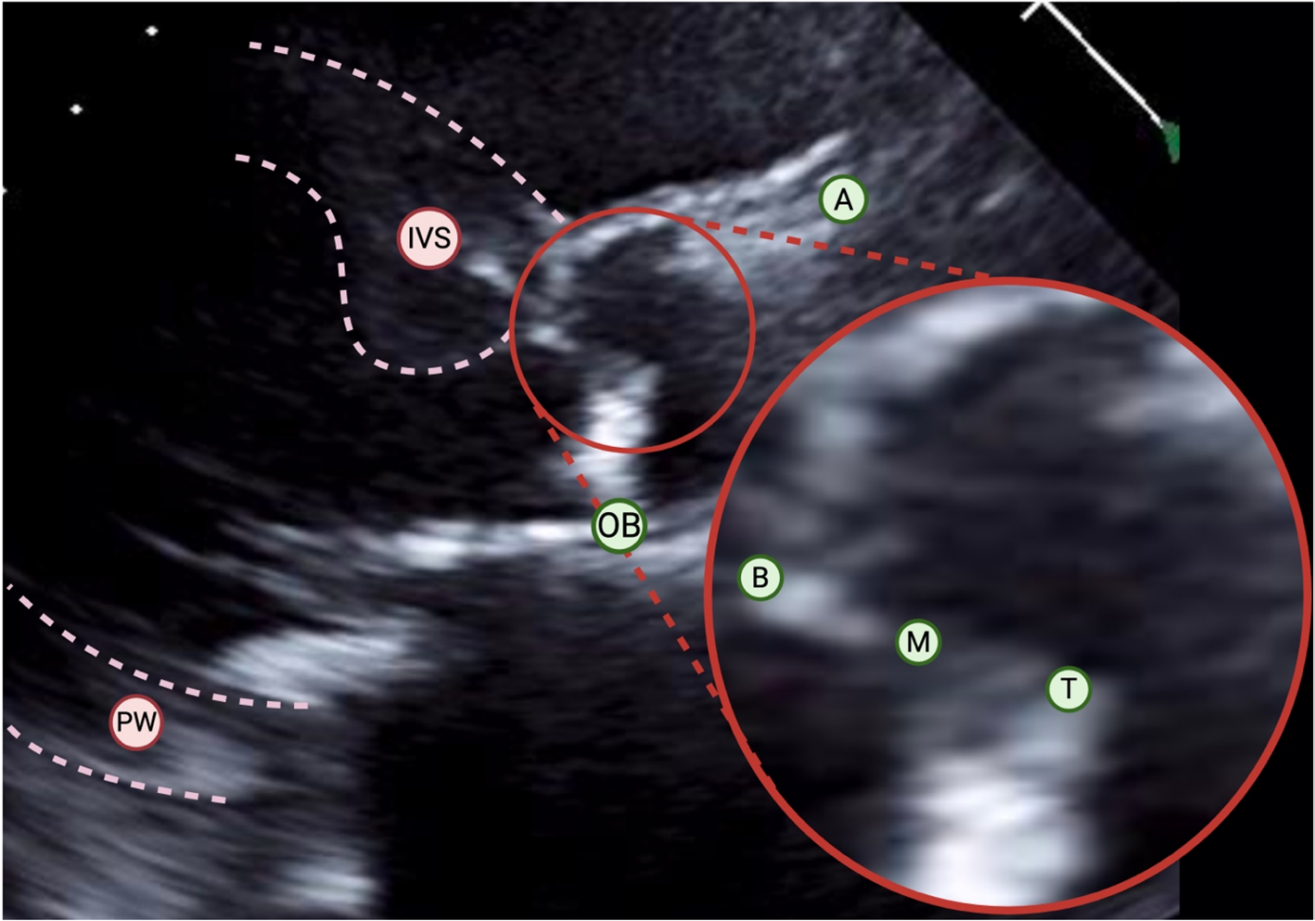
Parasternal long axis image with annotated landmarks. Three landmarks were created along the anterior (right coronary) leaflet (inset), in addition to the aortic wall and opposing leaflet hinge point, to calculate vectors. These vectors are then tracked in systole and diastole to generate motion data. A, aortic wall; B, base; IVS, interventricular septum; M, midpoint; OB, opposing base; PW, posterior wall; T, tip

The PLAX view is favorable for aortic leaflet analysis, as the angle of insonation is roughly perpendicular to the direction of aortic flow. In the standard plane, the more anterior leaflet corresponds to the right coronary cusp, and the posterior corresponds to the noncoronary cusp. The left coronary cusp is not traditionally visualized in the PLAX view but may appear in the position of the noncoronary cusp depending on the angle of insonation. Conversely, the right coronary cusp is always visualized as the upper leaflet, and therefore assessment of this leaflet is likely to be more consistent. As highlighted in Fig. 1, the following landmarks were specified: the leaflet base/hinge point, which is the interface between the base of the leaflet and the aortic root; the aortic wall at the level of the sinotubular junction; the mid segment of the right coronary leaflet; the right coronary leaflet tip; and the opposing leaflet hinge point (for determination of relative position and angular motion of the heart during the cardiac cycle).

### Vector allocation and dot-product calculation

**Fig. 2:**
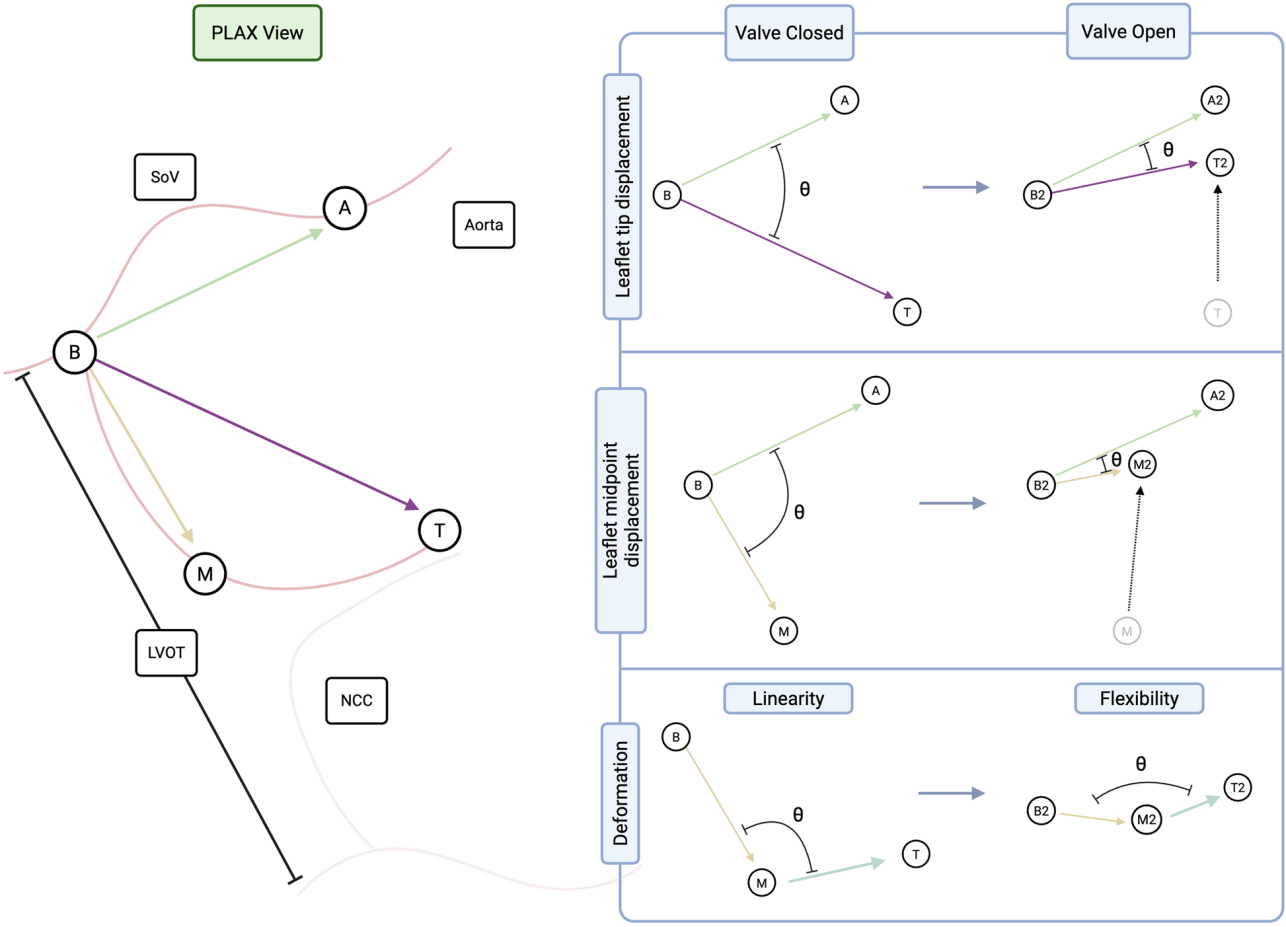
Schematic of leaflet landmarks and corresponding vectors and angles. Left panel: Illustration of the aortic valve as seen in parasternal long axis echocardiography. Right panel: Representation of vector and angle calculations for each landmark in open and closed states. Motion data in the form of Euclidean motion and angular rotation as well as flexibility were derived from these geometric data, as outlined in the Supplementary Materials. A, aortic wall landmark; B, leaflet base landmark; LVOT, left ventricular outflow tract; M, leaflet midpoint landmark; NCC, noncoronary cusp; SoV, sinus of Valsalva; T, leaflet tip landmark

Using the coordinates of these landmarks in both systole and diastole, and the pixel dimensions embedded in the DICOM (Digital Imaging and Communications in Medicine) metadata, it is possible to calculate vectors and corresponding Euclidean distances using linear algebra, as detailed in the Supplementary Material. Figure 2 illustrates the measured leaflet vectors. Vectors between the leaflet hinge point and the midpoint of the leaflet, the tip of the leaflet, the opposing leaflet hinge point, the aortic wall, and a “global” leaflet vector (representing the mean of the mid- and tip-point vectors) were generated. These five vectors were calculated in systolic (open) and diastolic (closed) phases, to derive motion data. We also quantified the intrinsic shape of the leaflet (linearity) by measuring the “internal” leaflet angle, with the leaflet midpoint acting as the apex of two vectors extending from this point to the tip, and to the leaflet hinge point. We postulated that the change in this angle, between the closed and open phases, might then reflect how dynamically flexible the leaflet is during motion.

### Statistical analysis and modelling

Continuous variables are presented as mean and standard deviations or median and interquartile range depending on normality. Categorical variables are presented as counts and percentages. Differences in metrics were compared using Kruskal–Wallis rank sum tests, given heteroscedasticity (unequal variance across classes). Post hoc identification of specific group differences was therefore performed with nonparametric Games-Howell pairwise testing, with Holm-Bonferroni adjustment for multiple comparison. Two predictive modelling tasks were performed with goals of classifying the binary outcome of “significant AS,” defined as moderate or worse AS, and specific severity classes, to distinguish normal, moderate, and severe classes from one another.

Three simple supervised machine learning classifiers were trained for these two predictive tasks: a logistic regression model with elastic net regularization, a k-nearest neighbors model, and a random forest model. There were no missing data. Data were split 80:20 into training and testing (holdout) sets, stratified by the outcome variable. The training set was subjected to fivefold cross validation for model tuning and evaluation. Significantly collinear variables (correlation threshold, > 0.9) were removed if present. Elastic net regularization was employed for the logistic regression models [9]. Model performance was assessed using standard metrics including area under the receiver operator characteristic (ROC) curve (AUC), sensitivity, specificity, positive predictive value (PPV; i.e., precision), and negative predictive value (NPV). Full model specifics, tuning parameters, and comparative approaches are outlined in the Supplementary Materials and Supplementary Table 1. Intraclass correlation coefficients were calculated on a random 10% sample to assess interrater and intrarater reliability testing. All analyses were performed with R version 4.4 (R Foundation for Statistical Computing).

## Results

### Baseline characteristics

The echocardiograms of 200 patients were included. Table 1 summarizes the baseline characteristics, Doppler-derived indices, and leaflet metrics of included patients. The mean age was 66 years and 86 (43%) were female. The mean AVA was 0.87 cm^2^ for severe cases, 1.20 cm^2^ for moderate cases, and 2.89 cm^2^ for control cases. Mean left ventricular ejection fractions were 57%, 62%, and 65%, for severe, moderate, and control cases, respectively. Frequency distributions of all novel leaflet metrics at each landmark are presented in Supplementary Fig. 1.

**Table 1:** Baseline characteristics

Characteristic	Overall (n = 200)	Control (n = 60)	Moderate AS (n = 70)	Severe AS (n = 70)	*P*-value^a^
Demographic					
Age (yr)	66 ± 22	39 ± 18	74 ± 9	80 ± 13	< 0.001
Sex					NS
Female	86 (43)	29 (48)	27 (39)	30 (43)	
Male	114 (57)	31 (52)	43 (61)	40 (57)	
Linear displacement (mm)					
Midpoint	3.4 ± 5.7	8.7 ± 3.1	3.1 ± 2.7	1.7 ± 1.6	< 0.001
Tip	4.5 ± 5.7	9.5 ± 3.7	4.2 ± 3.9	2.4 ± 2.1	< 0.001
Averaged	3.8 ± 5.5	9.2 ± 2.9	3.6 ± 3.1	2.1 ± 1.8	< 0.001
Angular displacement (°)					
Midpoint	22 ± 37	56 ± 14	21 ± 21	11 ± 12	< 0.001
Tip	19 ± 24	37 ± 13	17 ± 16	11 ± 10	< 0.001
Averaged	21 ± 30	47 ± 14	18 ± 18	11 ± 11	< 0.001
Deformation (°)					
Linearity	144 ± 33	117 ± 20	148 ± 26	159 ± 21	< 0.001
Flexibility	13 ± 35	52 ± 18	10 ± 17	5 ± 11	< 0.001
Standard metric					
Aortic valve area (cm^2^)	1.59 ± 0.97	2.89 ± 0.68	1.20 ± 0.36	0.87 ± 0.27	< 0.001
Mean gradient (mmHg)	23 ± 16	4 ± 1	23 ± 6	39 ± 11	< 0.001
Peak velocity (m/sec)	2.87 ± 1.16	1.30 ± 0.27	3.06 ± 0.39	3.97 ± 0.56	< 0.001
Dimensionless index	0.44 ± 0.26	0.80 ± 0.10	0.33 ± 0.11	0.24 ± 0.07	< 0.001
Left ventricular ejection fraction (%)	61 ± 12	65 ± 5	62 ± 11	57 ± 14	< 0.001

Values are presented as mean ± standard deviation or number (%)

AS, aortic stenosis; NS, not significant

^a^Kruskal-Wallis rank sum test and Pearson chi-square test

### Linear displacement

**Fig. 3:**
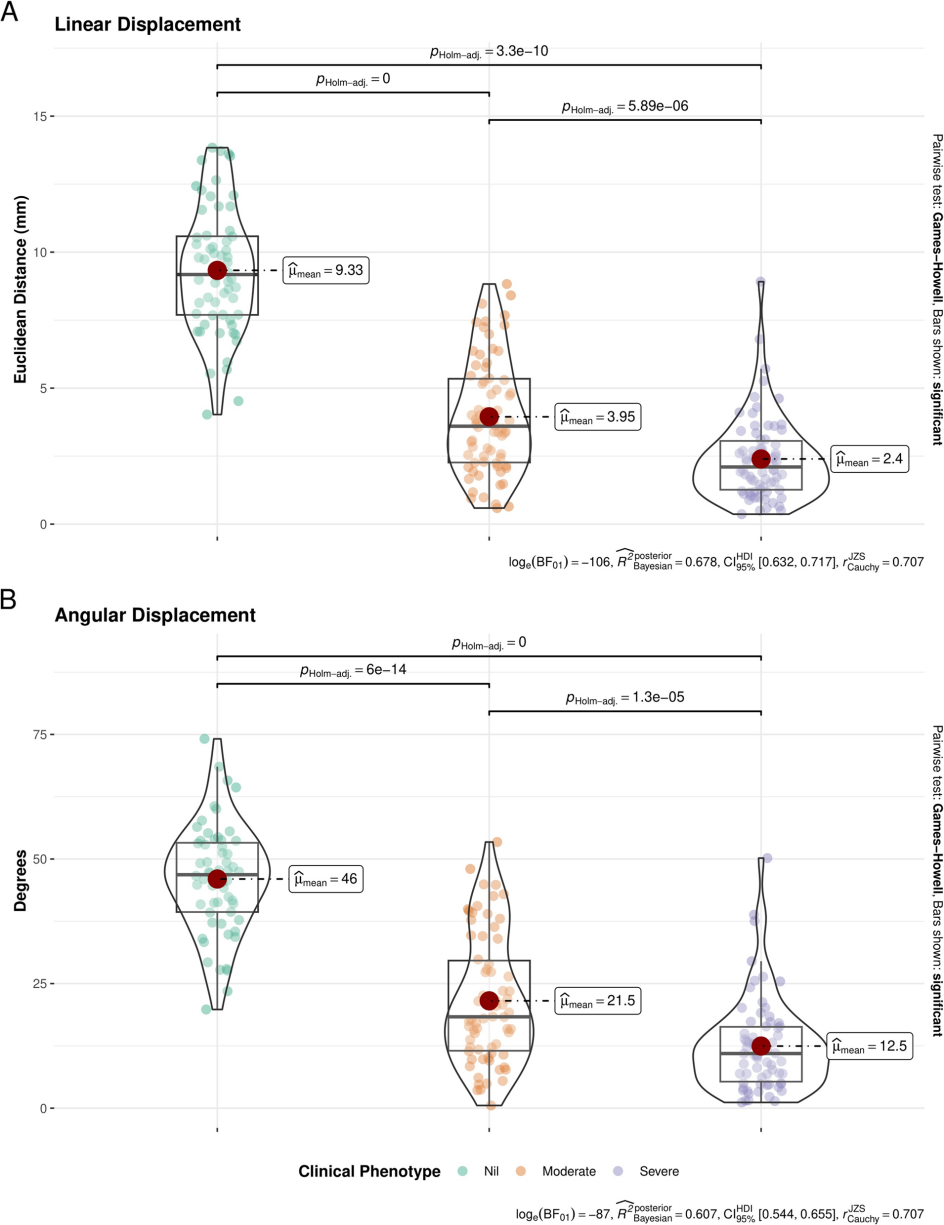
Comparison of global linear and angular displacement of leaflets between clinically adjudicated severity groups. **A** Violin plots for global linear displacement values. **B** Violin plots for global angular displacement values. There is significantly reduced linear and angular displacement for severe aortic stenosis, as compared with moderate and control cases

Linear displacement values, representing maximum distance travelled of individual leaflet components in millimeters, were significantly different across the severity spectrum of AS (Table 1). Between-class analysis revealed that severe cases demonstrated significantly reduced linear displacement, at each landmark (Supplementary Fig. 2) compared with moderate and normal cases. Pairwise comparisons of global linear displacement are presented in Fig. 3A. Severe cases demonstrated averaged (global) leaflet motion of 2.1 mm, compared with 3.6 mm for moderate cases (*P* < 0.01) and 9.2 mm for control cases (*P* < 0.01). A similar pattern was observed when cases were grouped by Doppler hemodynamic criteria (Supplementary Table 3); there was a trend towards reduced linear displacement in high-gradient severe AS versus low-gradient severe AS, although this was not statistically significant. However, hemodynamically defined moderate cases were statistically distinguished from high-gradient severe AS cases at each landmark (Supplementary Fig. 3).

### Angular displacement

Angular displacement values, representing rotation of individual leaflet components with respect to the aortic wall, in degrees, were significantly different across the severity spectrum of AS (Supplementary Fig. 4). Severe cases demonstrated mean global angular rotation of 11°, which was significantly less than moderate (18°, *P* < 0.01) and control cases (47°, *P* < 0.01) (Fig. 3B). When grouped by hemodynamic criteria, a similar inverse correlation between angular rotation and advancing severity of AS was observed (Supplementary Fig. 5). Again, when grouped by hemodynamic criteria, low- and high-gradient AS were indistinguishable with respect to angular rotation, though there was a trend to reduced rotation in high-gradient cases.

### Leaflet shape and deformation

In diastole, control (normal) leaflets demonstrate significantly less linearity, (i.e., greater concavity) consistent with the natural curvilinear anatomy of a healthy leaflet (Supplementary Fig. 6). Control leaflets demonstrated an internal resting (diastolic) angle of 117°, significantly less than severe cases, which demonstrated much greater linearity with advancing calcification (159°, *P* < 0.01). During motion, normal leaflets demonstrate significantly greater internal flexibility (absolute change in internal angle, 52°), compared to moderate (10°, *P* < 0.01) and severe cases (5°, *P* < 0.01), reflecting greater rigidity as disease progresses.

### Correlation and reproducibility

As presented in Supplementary Fig. 7, both linear and angular displacement correlate positively with calculated AVA (r = 0.81 and r = 0.74, respectively), suggesting that leaflet motion deteriorates with decreasing AVA, as expected. Linear displacement appeared to correlate better than angular displacement. The mean intraclass correlation coefficients for interrater and intrarater reliability analysis were 0.858 (95% confidence interval [CI], 0.68–0.94) and 0.831 (95% CI, 0.63–0.93), respectively, reflecting very good agreement. Individual intraclass correlation coefficients for each metric are presented in Supplementary Tables 3 and 4.

### Classification performance

#### Task 1: Predicting “significant” disease as a binary outcome

**Fig. 4:**
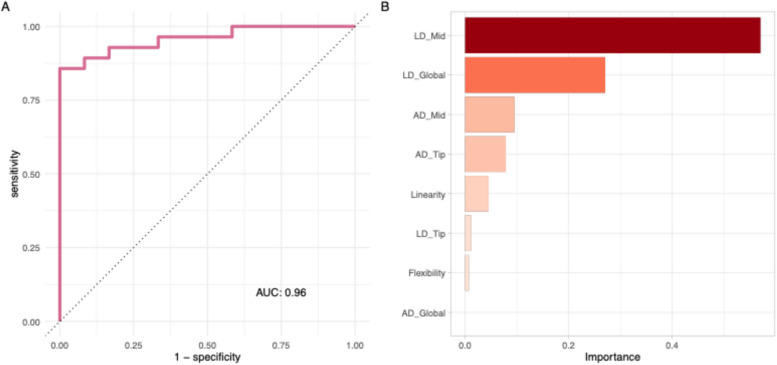
Performance of the best performing model for task 1 (linear regression with elastic net regularization). **A** The receiver operator characteristic (ROC) curves for the multinomial linear regression with elastic net regularization model on the holdout test data. Each curve represents the distinction between that severity class against the remaining two classes. The mean (global) area under the ROC curve (AUC) is 0.96. **B** The features ranked in descending order of input importance; mid-leaflet linear displacement is the most important feature. AD, angular displacement; LD, linear displacement

Three separate supervised-learning models were trained to discriminate “significant AS,” being moderate or greater disease, from controls. The AUCs for the logistic regression with elastic net regularization, random forest, and k-nearest neighbors models were 0.98, 0.96, and 0.97, respectively. The corresponding ROC curves and AUC comparisons are demonstrated in Supplementary Fig. 8. Model performance was not significantly different between approaches (P = 0.81). For simplicity and maximum explainability, the elastic net logistic regression model was selected for final model evaluation on the holdout test data. Logistic regression with elastic net regularization predicted significant AS with an accuracy of 90% and AUC of 0.96. The sensitivity and specificity were 93% and 83%, respectively. The PPV (precision) and NPV were 92.9% and 83.3%, respectively. Figure 4 illustrates the ROC curve and relative importance of predictive features in the final model.

#### Task 2: Discriminating individual severity classes

**Fig. 5:**
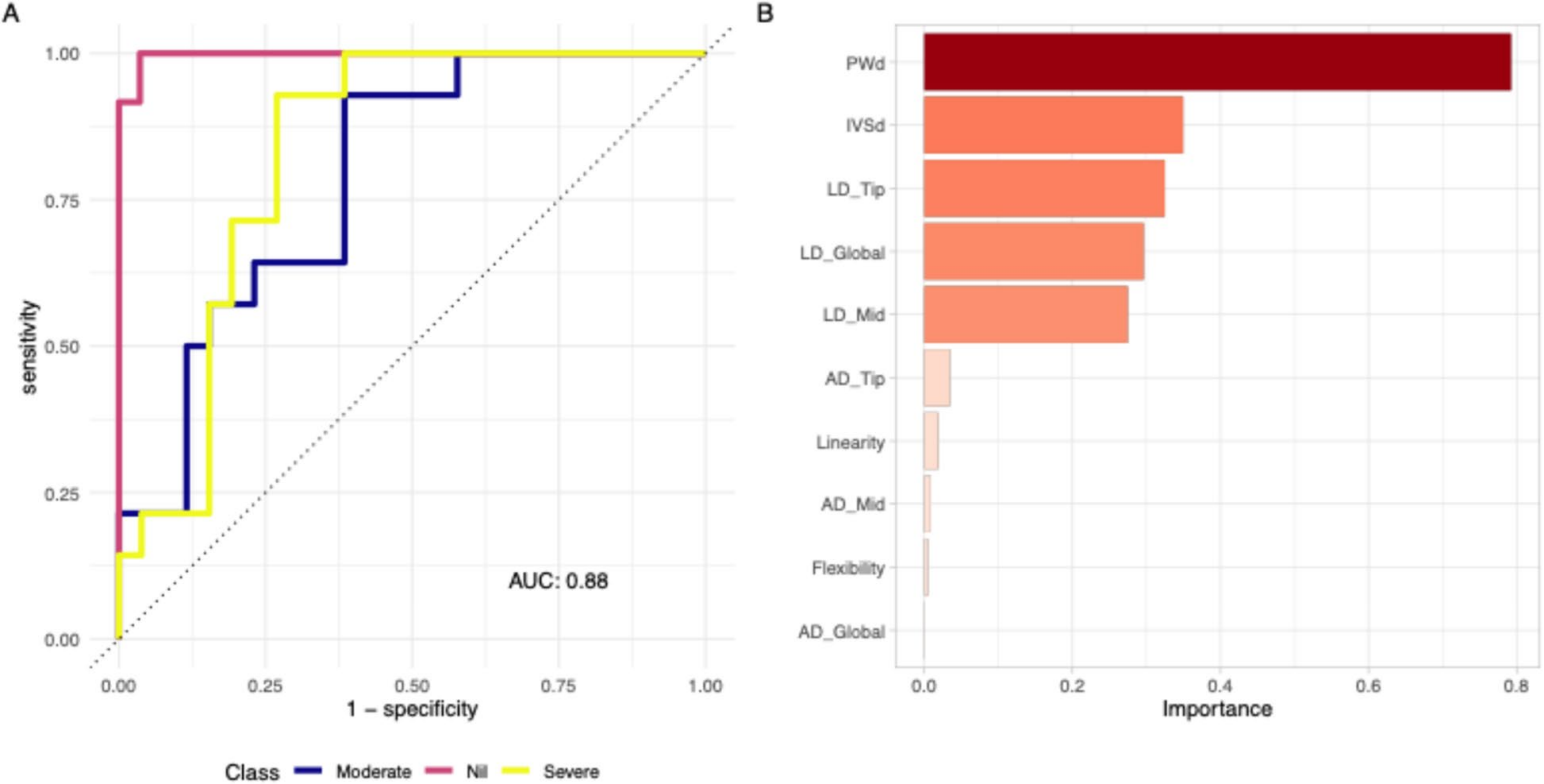
Performance of the best performing model for task 2: multinomial linear regression with elastic net regularization. **A** The receiver operator characteristics (ROC) curve for the linear regression with elastic net regularization model on the hold-out test data. The area under the ROC curve (AUC) is 0.88. **B** The features ranked in descending order of input importance. AD, angular displacement; IVSd, interventricular septal diameter; LD, linear displacement; PWd, posterior wall diameter

The second classification task was to accurately distinguish individual severity classes. We deployed the same models with adjustments to handle the multiclass outcome; multinomial logistic regression model with elastic net regularization, a random forest, and k-nearest neighbors. Owing to the difficulty in discriminating moderate from severe cases, two additional metrics routinely captured from the PLAX view were added as predictor variables: the interventricular septal and posterior wall diameters. The AUCs for the multinomial logistic regression with elastic net regularization, random forest, and k-nearest neighbors models were 0.88, 0.88, and 0.9, respectively. Again, no single model was statistically superior to another (P = 0.56) (Supplementary Fig. 9), but for simplicity the multinomial logistic regression model was selected for exposure to the test data. The final model demonstrated the following global (mean) performance metrics: accuracy, 72.5%; sensitivity, 73.8%; specificity, 86%; PPV, 73%; NPV, 86.9%; and AUC, 0.88. Figure 5 illustrates the final ROC curves and relative importance of predictive features in the final model.

## Discussion

We present a simple and accurate AS classifier using a single PLAX view, without any need for hemodynamic or Doppler assessment. Our model, grounded in biological plausibility, simple linear algebra and supervised machine learning, provides a highly explainable approach to disease identification. We have demonstrated that aortic leaflet motion and AS-related restriction can be accurately quantified using a single two-dimensional (2D) PLAX echocardiographic view. With increasing severity of AS, there is an associated reduction in echocardiographically quantified leaflet motion. We also quantified the loss of convexity (increased linearity) in leaflet morphology as AS progresses. Using these novel metrics, simple supervised machine learning classification demonstrated impressive predictive power. Specifically, these metrics can almost perfectly predict significant AS (moderate or worse) from the PLAX view alone. Discriminating moderate from severe disease is a more difficult task with less, though still impressive, predictive accuracy.

Direct echocardiographic quantification of leaflet restriction is not a novel concept, and indeed garnered significant research interest in the late 1970s. In 1975, Weyman et al. [10] published a series of 28 patients with AS, upon whom aortic orifice dimensions were recorded using cross-sectional and M-mode echocardiography. They eloquently illustrated the inverse correlation with 2D aortic orifice dimension and invasively adjudicated stenotic severity, highlighting significant differences between severity groups. Although the presence of abnormal motion was shown to be highly sensitive for the presence of any degree of AS, subsequent groups failed to identify meaningful correlations between invasively derived metrics and leaflet excursion, likely due to limitations in sample size, imaging resolution, measurement feasibility, as well as selection of patients with very advanced disease by today’s standards [11, 12].

The recent application of deep learning approaches to single view echocardiography has facilitated use of features embedded within 2D PLAX images, with incredible predictive power, albeit without clear explainability or identification of the exact nature of the features. Wessler and colleagues recently reported high classification accuracy of AS severity classes by applying convolutional neural network classifiers to single PLAX still frames [4]. Saliency heatmapping can be employed to help understand what imaging features are contributing to model decisions, and studies have demonstrated that models are paying attention to the aortic valve region [7, 13], as expected, but extracting quantifiable features from these convolutional neural networks is not currently possible. These regions of interest have interestingly been labelled “hypothesis-generating.” Our findings directly support the foundational work of Weyman et al. [10] and provide complementary explainability to recent deep learning-based observations, highlighting the merit in leaflet motion quantification.

We selected basic, robust, and common supervised machine learning models for our classification tasks to maximize explainability and reproducibility. The performance of each model was not materially different, and so logistic regression was chosen for exposure to the test set to maintain simplicity. We were intrigued by the variable importance rankings, which highlighted greater weighting of linear displacement, rather than angular displacement. Moreover, the width of the interventricular septum and posterior left ventricular walls were highest ranking in the subclassification model, highlighting the intricate link between AS severity and left ventricular hypertrophy. It is important to note that these are relative weightings, and that all features contributed to final performance.

There are several implications of our work. First, our approach could feasibly be translated to point-of-care ultrasound, which would facilitate screening for significant AS by non-cardiac sonographers. This would require very little computational power, compared to complex deep learning approaches. This could be of great advantage to under-serviced rural or lower socioeconomic populations, for whom dedicated echocardiography may not be easily accessible. In general, nondedicated ultrasound technology is widely available, inexpensive, safe and reproducible. However, accurate interpretation of the echocardiogram requires years of training, and accurate classification of AS remains a perpetual challenge to even experienced cardiologists. Based on these foundational data, further research into leaflet mechanics under specific flow conditions is necessary to understand true leaflet restriction and accurate disease classification. These results set the stage for further development of automated and explainable echocardiographic techniques to improve classification speed and accuracy.

### Limitations

Several limitations warrant attention. Our sample size was small, but was sufficiently powered to determine statistical differences in leaflet metrics between major severity classes. We employed robust statistical techniques to prevent model overfitting, however external validation of this work on a larger sample size is required, and is currently underway. Owing to this sample size, accurate classification of different flow-gradient subgroups was not possible. Our cohort also did not include participants with bicuspid or rheumatic AS to determine disease-specific predictive ability. Conceptually these populations could be identified using this approach, with some important considerations. It is well known that AS can asymmetrically involve the three leaflets, particularly in the presence of abnormal calcium handling and comorbid renal disease. Therefore, quantifying motion of only the right coronary leaflet introduces a degree of error, and may overestimate or underestimate AS severity in these subgroups. However, the right coronary leaflet is the most identifiable leaflet on PLAX assessment, as the more posterior leaflet can represent either the left or noncoronary leaflet depending on the angle of insonation. Moreover, the posterior leaflet is often shadowed by the calcified right coronary leaflet in advanced disease. Incorporation of motion data from the short-axis view or 3D imaging may be a step towards improving accuracy in these subgroups affected by asymmetric leaflet calcification. Investigation of leaflet motion in these subgroups is currently planned. Owing to limitations in spatial and temporal resolution of echocardiography, quantification of leaflet motion will be accompanied by a margin of error, which in part contributes to the variance of observed values for each severity class. Improved accuracy might be observed with alternate imaging modalities such as magnetic resonance imaging; however, this is rarely performed for investigation of AS since echocardiography is the gold-standard modality for diagnosis.

## Conclusions

We present a simple method to quantify aortic leaflet motion. These motion data can accurately classify AS using a single parasternal long axis view, without the need for hemodynamic or Doppler assessment. Our model, grounded in biological plausibility, simple linear algebra and supervised machine learning, provides a highly explainable approach to disease identification and may hold significant clinical utility for the diagnosis and classification of AS.

## Supplementary Information

Supplementary Material 1.

## Data Availability

Data are available upon reasonable request to the authors.

## Abbreviations

2D: Two-dimensional
AS: Aortic stenosis
AUC: Area under the receiver operator characteristic curve
AVA: Aortic valve area
CI: Confidence interval
DICOM: Digital Imaging and Communications in Medicine
NPV: Negative predictive value
PLAX: Parasternal long axis
PPV: Positive predictive value
ROC: Receiver operator characteristic
