# Supplementary material for: Aortic valve leaflet motion for diagnosis and classification of aortic stenosis using single view echocardiography
Source: J Cardiovasc Imaging. 2025 Jul 8;33:8. doi: 10.1186/s44348-025-00051-8 (PMC12235788; doi:10.1186/s44348-025-00051-8)
Supplement: Supplementary file 1 — Supplementary Material 1. [file 44348_2025_51_MOESM1_ESM.docx]

**Supplemental Material**

**Title:** Aortic valve leaflet motion for novel, rapid and accurate and classification of aortic stenosis using single view echocardiography

**Authors:**

Thomas Meredith (BMed MD MClinTRes) ^a,b,c^

Farhan Mohammed (PhD)^b^

Amy Pomeroy (DipCardiacUS)^b^

Sebastiano Barbieri (PhD)^e,d^

Erik Meijering (MSc PhD)^c^

Louisa Jorm (MSc PhD)^d^

David Roy (MBChB)^a^

Christopher Hayward (BMed MD)^a,b,c^

Jason C. Kovacic (MBBS PhD)^a,b,c,d^

David W.M. Muller (MBBS MD)^a,b^

Michael Feneley (MD PhD)^a,b,c^

Mayooran Namasivayam (MBBS PhD) ^a,b,c^

^a^ Department of Cardiology, St Vincent’s Hospital, Sydney, Australia
^b^ Victor Chang Cardiac Research Institute, Sydney, Australia

^c^ University of New South Wales, Sydney, Australia

^d^ Centre for Big Data Research in Health, University of New South Wales, Sydney, Australia

^e^ Icahn School of Medicine at Mount Sinai, New York, NY, USA

^f^ Queensland Digital Health Centre, University of Queensland, Brisbane, Australia

Table of Contents

[Supplemental Methods 2](#_Toc198665542)

[Supplemental Tables 4](#_Toc198665543)

[Supplemental Table 1 4](#_Toc198665544)

[Supplemental Table 2 4](#_Toc198665545)

[Supplemental Table 3 4](#_Toc198665546)

[Supplemental Table 4 4](#_Toc198665547)

[Supplemental Figures 6](#_Toc198665548)

[Supplemental Figure 1 6](#_Toc198665549)

[Supplemental Figure 2 8](#_Toc198665550)

[Supplemental Figure 3 9](#_Toc198665551)

[Supplemental Figure 4 10](#_Toc198665552)

[Supplemental Figure 5 11](#_Toc198665553)

[Supplemental Figure 6 12](#_Toc198665554)

[Supplemental Figure 7 13](#_Toc198665555)

[Supplemental Figure 8 14](#_Toc198665556)

[Supplemental Figure 9 15](#_Toc198665557)

[References 16](#_Toc198665558)

# Supplemental Methods

*Metric calculation*

Vectors between the leaflet hinge-point and a) the midpoint of the leaflet, b) the tip of the leaflet, c) the opposing leaflet hinge-point, d) the aortic wall, and e) a ‘global’ leaflet vector (representing the mean of the mid-point and tip-point vectors) were generated. Vectors are calculated by subtracting the x- and y-coordinates of one landmark from another. Since a vector represents both magnitude and angle/direction, knowledge of one component facilitates derivation of the other. The *magnitude* of a vector (in our case, the distance between two landmarks) can be determined by calculating the square root of the sum of the square of its components. By using the pixel dimensions embedded in the DICOM metadata, one can convert the landmark coordinates from pixel format to distance (mm) format, and then determine the distances individual landmarks travel between timepoints (systole and diastole). The magnitude (length) of vector spanning A to B can then be reverse calculated. To determine leaflet motion *independent* from overall cardiac motion, the leaflet landmark coordinates were referenced to the hinge point. To determine angular displacement between leaflet landmarks and the aortic root, one can calculate the dot product, which is a scalar output indicating alignment between two or more vectors.^1^ The angle between two vectors can subsequently be determined, as the dot product is also equal to the product of both vector magnitudes, multiplied by the cosine of the angle between them. Therefore, with knowledge of the dot product and vector magnitudes, the cosine of the angle can be deduced. The angle can then be calculated using the inverse cosine function.

*Doppler hemodynamic subgroups*

As per ASE criteria, high-gradient severe AS was defined by a mean gradient >40mmHg and AVA <1.0cm^2^. Low-gradient severe AS was defined by a mean gradient ≤40mmHg and AVA < 1.0cm^2^. Moderate AS was defined by a mean gradient ≤40mmHg and ≥20mmHg and AVA ≥1.0cm2. Mild was defined mean gradient <20mmHg and max velocity >2.5m/s, and no significant AS by max velocity ≤2.5m/s.

*Model Specifications*

Tuning for all models was performed using the *dials* package in R.^2^ For elastic net regularization, a 20x20 tuning grid was generated, comprising a total of 400 combinations of randomly initialized penalties and mixtures. The penalty pertains to the extent that coefficients are shrunk (to avoid overfitting), and the mixture controls the balance between the two types of penalties: Lasso (L1) ad Ridge (L2). Tuning grids were also generated for random forest parameters (number and depth of trees) and knn parameters (number of neighbors). The best tuning parameters for each model were selected through cross validation and identification of best AUC and are reported in Supplemental Table 1. Predictors were assessed for collinearity (correlation >0.9), which was not identified. Normalization of predictor variables was performed for the k-nearest neighbors model.

*Model Comparison*

Normality of cross-validated (training) AUC values was determined using Shapiro Wilk tests for each model. In the presence of normality, ANOVA was performed to determine a generalized difference, prior to direct between-model comparison with Tukey’s range test.

# Supplemental Tables

## Supplemental Table 1

| Classifier | Task 1 Parameters | Task 2 Parameters |
| --- | --- | --- |
| Logistic Regression | Penalty = 0.0264, Mixture = 0.65 | Penalty = 0.298, Mixture = 0.053 |
| Random Forest | 500 trees, 2 nodes | 387 trees, 2 nodes |
| K-nearest neighbor | K = 12 | K = 16 |

## Supplemental Table 2

| **Group** | **Characteristic** | **Overall**, N = 192 | **Nil**, N = 58 | **Mild**, N = 19 | **Moderate**, N = 34 | **Severe - LG**, N = 49 | **Severe - HG**, N = 32 | **p-value** |
| --- | --- | --- | --- | --- | --- | --- | --- | --- |
| Linear Displacement (mm) | Midpoint | 4.6 (3.5) | 9.0 (2.5) | 3.9 (2.0) | 3.6 (2.1) | 2.3 (1.5) | 1.9 (1.2) | **<0.001** |
|  | Tip | 5.4 (3.6) | 9.5 (2.8) | 4.4 (2.9) | 4.6 (2.4) | 3.2 (2.0) | 2.8 (1.7) | **<0.001** |
|  | Averaged | 5.0 (3.5) | 9.3 (2.5) | 4.2 (2.4) | 4.1 (2.1) | 2.7 (1.7) | 2.4 (1.3) | **<0.001** |
| Angular Displacement (deg) | Midpoint | 30 (21) | 55 (13) | 26 (14) | 24 (16) | 16 (12) | 13 (10) | **<0.001** |
|  | Tip | 22 (15) | 37 (11) | 20 (12) | 19 (12) | 14 (10) | 12 (9) | **<0.001** |
|  | Averaged | 26 (18) | 46 (11) | 23 (13) | 22 (13) | 15 (11) | 12 (9) | **<0.001** |
| Deformation | Linearity | 143 (21) | 120 (14) | 141 (14) | 146 (18) | 156 (14) | 161 (11) | **<0.001** |
|  | Flexibility | 22 (22) | 48 (18) | 16 (10) | 13 (16) | 11 (10) | 6 (6) | **<0.001** |

## Supplemental Table 3

| Metric | ICC | Lower 95% CI | Upper 95% CI | p-value |
| --- | --- | --- | --- | --- |
| LD_Tip | 0.8198742 | 0.5999224 | 0.9246012 | <0.01 |
| LD_Mid | 0.8756984 | 0.7131697 | 0.9488803 | <0.01 |
| LD_Global | 0.8582285 | 0.6767934 | 0.9413743 | <0.01 |
| AD_Tip | 0.9094993 | 0.7861271 | 0.9631718 | <0.01 |
| AD_Global | 0.9004623 | 0.7662787 | 0.9593804 | <0.01 |
| Flexibility | 0.7395023 | 0.4510138 | 0.8880847 | <0.01 |
| Linearity | 0.7146982 | 0.4080876 | 0.8764232 | <0.01 |

## Supplemental Table 4

| Metric | ICC | Lower 95% CI | Upper 95% CI | p-value |
| --- | --- | --- | --- | --- |
| LD_Tip | 0.7606870 | 0.4887646 | 0.8978954 | <0.01 |
| LD_Mid | 0.9199874 | 0.8094863 | 0.9675456 | <0.01 |
| LD_Global | 0.8543787 | 0.6688947 | 0.9397091 | <0.01 |
| AD_Tip | 0.7952521 | 0.5526252 | 0.9136151 | <0.01 |
| AD_Global | 0.8557977 | 0.6718011 | 0.9403233 | <0.01 |
| Flexibility | 0.9361119 | 0.8460944 | 0.9742147 | <0.01 |
| Linearity | 0.8853290 | 0.7336043 | 0.9529831 | <0.01 |

# Supplemental Figures

## Supplemental Figure 1

Title: Density plots of novel leaflet motion indices

Caption: Density plots reveal the distribution of recorded leaflet motion values across severity classes. The red lines indicated pooled frequency values. Panel A demonstrates plots for linear displacement at each leaflet landmark; Panel B demonstrates angular displacement; Panel C demonstrates deformation distributions.

## Supplemental Figure 2

Title: Linear displacement across reporter-adjudicated severity groups

Caption: With advancing severity of AS, there is an associated reduction in linear displacement. Panel A demonstrates global (averaged) values. Panel B demonstrates values for the midpoint and Panel C demonstrates values for the leaflet tip. At each landmark, there is a significant between-class difference in linear displacement.

## Supplemental Figure 3

Title: Linear displacement across Doppler hemodynamic severity groups

Caption: With advancing severity of AS, there is an associated reduction in linear displacement. Panel A demonstrates global (averaged) values. Panel B demonstrates values for the midpoint and Panel C demonstrates values for the leaflet tip.

## Supplemental Figure 4

Title: Angular displacement across reported adjudicated severity groups

Caption: With advancing severity of AS, there is an associated reduction in angular displacement. Panel A demonstrates global (averaged) values. Panel B demonstrates values for the midpoint and Panel C demonstrates values for the leaflet tip. At each landmark, there is a significant between-class difference in angular displacement.

## Supplemental Figure 5

Title: Angular displacement across Doppler hemodynamic severity groups

Caption: With advancing severity of AS, there is an associated reduction in angular displacement. Panel A demonstrates global (averaged) values. Panel B demonstrates values for the midpoint and Panel C demonstrates values for the leaflet tip.

## Supplemental Figure 6

Title: Comparison of deformation indices across severity grades.

Caption: Healthy curvilinear leaflets demonstrate less linearity in the resting state (Panel A) and greater flexibility during motion (Panel B).

## Supplemental Figure 7

Title: The correlation between leaflet motion and aortic valve area (AVA).

Caption: Scatterplots comparing linear displacement (Panel A) and angular displacement (Panel B) with aortic valve area. Linear regression lines are plotted in red, demonstrating strong positive correlation between AVA and both linear displacement (R = 0.81) and angular displacement (0.74).

## Supplemental Figure 8

Title: Performance of task 1 models: distinguishing significant from non-significant AS.

Caption: Panel A illustrates the ROC curves for each classification model. Panel B illustrates the comparison of cross-validated AUC results for each model. There was no significant difference between model performance.

## Supplemental Figure 9

Title: Performance of task 2 models: distinguishing individual severity grades.

Caption: Panel A illustrates the ROC curves for each classification model and severity grade. Panel B illustrates the comparison of cross-validated AUC results for each model. There was no significant difference between model performance.

# References

1. Strang G. *Introduction to Linear Algebra*. 6th ed. Cambridge University Press, 2023.

2. Kuhn M, Frick H. *dials: Tools for Creating Tuning Parameter Values*. 2024.
